# Church-Going

**Published:** 1872-10

**Authors:** 


### CHURCH-GOING.

THE almost incredible statement is made, that in the beautiful city of Berlin, with its million of inhabitants, only about twelve thousand attend church on Sundays. This neglect of the worship of the Almighty on his own appointed day is one of the natural results of Sunday concerts and open beer-gardens, and other amusements, on the Sabbath-day, and it is to be feared that the growing tendencies to keep public libraries and reading-rooms open on Sunday will have the effect to win young men from the services of the sanctuary.

The night is the most dangerous time for young men in cities; darkness covers wrong-doing, and invites to ways that lead to ruin, to idleness, drunkenness, and crime; hence it might answer a good purpose to keep reading-rooms open from sundown to ten o'clock on Sunday nights. At the same time, any young man who considers it his duty to spend the Sabbath-day at home, or at church, or in visiting the poor, is worthy of the respect and encouragement of all good people. Such young men do not smoke cigars, loiter on the streets, visit theatres, and drink whiskey.

